# Reliability and Validity of Non-radiographic Methods of Forward Head Posture Measurement: A Systematic Review

**DOI:** 10.7759/cureus.27696

**Published:** 2022-08-05

**Authors:** Konstantinos Mylonas, Maria Tsekoura, Evdokia Billis, Pavlos Aggelopoulos, Elias Tsepis, Konstantinos Fousekis

**Affiliations:** 1 Department of Physiotherapy, Laboratory of Therapeutic Exercise and Sports Rehabilitation, University of Patras, Aigio, GRC

**Keywords:** craniovertebral angle, neck pain, non-radiographic methods, measurement, validity, reliability, forward head posture

## Abstract

Forward head posture measurement can be conducted using various methods and instruments. The selection of the appropriate method requires the factors of validity and reliability to be considered. This systematic review reports on the reliability and validity of the non-radiographic methods examined for measuring forward head posture. The review identified relevant studies following a systematic search of electronic databases. The studies were assessed for quality by two independent reviewers using a critical appraisal tool. The studies’ data were extracted and assessed, and the results were synthesized qualitatively using a level of evidence approach. Twenty-one studies met the eligibility criteria and were included in the review. Both reliability and validity were investigated for five studies, whereas reliability only was investigated for 17 studies. In total, 11 methods of forward head posture measurement were evaluated in the retrieved studies. The validity of the methods ranged from low to very high. The reliability of the methods ranged from moderate to excellent. The strongest levels of evidence for reliability support the use of classic photogrammetry. For validity, the evidence is not conclusive. Further studies are required to strengthen the level of evidence on the reliability and validity of the remaining methods. It is recommended that this point be addressed in future research.

## Introduction and background

Neck pain shows high epidemiological occurrence [1,2]. According to the Bone and Joint Decade 2000-2010 Task Force on Neck Pain and Its Associated Disorders, most people experience some neck pain in their lifetimes [3]. However, for most, the pain does not seriously hinder everyday activities. At least one in three adults in Europe and North America experiences neck pain at some point. About 5-10% of these cases involve severe neck pain. The prevalence of neck pain is higher in women, and it increases with age [1,4,5]. Additional risk factors are lack of physical activity, increased body mass index (BMI), low kinaesthesia, and incorrect movement patterns [6-9]. Neck pain has also been associated with poor health, previous neck injuries and other risk factors, including occupation, smoking and obesity and bad posture [3,10-12].

The most common pathological postural adaptation associated with neck pain development is the forward head posture (FHP) [8,13]. FHP increases weight pressure on the cervical spine, enhancing pathological myofascial adaptations and muscle imbalances. Amongst others, the muscles that FHP weakens include the deep neck flexors, scapular stabilizers, and retractors. The muscles that become shortened and overactive include the deep upper cervical extensors, shoulder protractors, and elevators. Those muscle imbalances can cause cervical and thoracic instability, resulting in decreased respiratory function, proprioceptive alterations, increased muscle tone and cervical pain [14,15].

The anterior displacement of the head is mainly assessed through examination of the craniovertebral angle (CVA) as defined by Wickens and Kipputh [16]. CVA measurement is essential to the musculoskeletal assessment, helping clinical therapists screen for excessive anterior head displacement and develop correct therapeutic strategies for this pathological condition.

The current gold standard for the quantitative determination of the cervical angle is the lateral x-ray, which, however, shows significant limitations in its use such as the high cost of examination and exposure of patients to high doses of potentially harmful radiation. Alternatively, several non-invasive examination methods have been adopted for clinical use, including imaging-photographs, goniometry, and three-dimensional (3D) motion devices [17-21]. Guidelines for selecting assessment tools in clinical and laboratory testing settings recommend that the validity and reliability of measurement tools are among the key parameters to be ensured [22-24]. Validity refers to the truth of a set of statements [25,26], and it examines whether a study instrument measures the variable it intends to measure [22,26]. In contrast, reliability is the reproducibility of results upon repeated trials [26,27] without error.

Since several studies have been published on the validity and reliability of CVA non-invasive screening tools, a literature review is needed to draw conclusions and provide valuable clinical guidelines. Therefore, the purpose of this systematic review was to report on the reliability and validity of non-radiographic methods of measuring FHP.

## Review

Methods

Search Strategy

The primary investigator conducted a systematic search from April 1 to May 1, 2022. Databases included PubMed, MEDLINE (Medical Literature Analysis and Retrieval System Online), EBSCO (Elton B. Stephens Company), Google Scholar, and Science Direct. The keywords used in different combinations were: forward head posture, craniovertebral angle, test, measurement, validity, reliability, cervical photogrammetry and radiography.

After the initial search, duplicate articles were removed, and the remaining studies were assessed based on the title and abstract. The full-text article was searched and analyzed when the article appeared to meet the inclusion criteria. A full reading of the articles was then conducted to ensure relevance, and seven articles were removed. The reference lists of the articles were further searched for additional articles, but none were identified as relevant.

Eligibility Criteria

The eligibility criteria were agreed upon during a meeting between the two reviewers. The inclusion criteria were as follows: 1) articles available in full text, 2) articles available in the English and Greek language, 3) FHP recorded with non-invasive techniques, 4) included measurement of validity and/or reliability, and 5) human participants being part of the study, with no restrictions on their physical and somatic characteristics. The exclusion criteria were as follows: 1) radiographic measurement techniques only, and 2) no intraclass correlation coefficient (ICC) calculated for the measurement of reliability. When the final list of articles was drafted, the secondary reviewer checked it across the eligibility criteria. No disagreements occurred between reviewers regarding the eligibility of chosen articles.

Quality Assessment

The reviewers used the checklist by Brink and Louw (2011), representing a critical appraisal tool [28]. This was designed to assess the methodological quality of studies by testing the validity and reliability of objective clinical tools. The checklist comprises 13 questions that qualitatively assess the methodology of studies by combining the Quality Assessment of Diagnostic Accuracy Studies (QUADAS) [29] and the Quality Appraisal of Diagnostic Reliability Studies (QAREL) [30]. Response options for the 13 questions are ‘yes’, ‘no’, or ‘N/A’ (not applicable). This checklist was also used in a systematic review of non-radiographic thoracic kyphosis measurements [31]. In that systematic review, Barrett et al. included articles, some of which assessed both reliability and validity [31]. Therefore, this checklist was deemed more convenient than using the QUADAS or QUAREL separately. The studies were awarded a high-quality score if a positive score (‘yes’) was given to 60% or more of the questions (≥60%); the same scheme was used previously by van der Wurff et al. [32], May et al. [33], and Adhia et al. [34].

Quality assessment was first performed by the primary reviewer. In the next stage, the secondary reviewer checked the rating of the primary reviewer. Limited differences arose; these were discussed based on the proper interpretation of questions and which response would more accurately reflect the reality. No kappa score was recorded because there were very few diverging views and consensus was quickly reached.

Data Analysis

The collected studies showed large heterogeneity in the study populations and measurement tests. Therefore, neither meta-analysis nor subgroup analysis was deemed feasible. Consequently, the reviewers performed a descriptive analysis by synthesising data using the evidence approach [35], as shown in Table 1.

**Table 1:** Levels of evidence approach

Level of evidence	Criteria
Strong	Consistent findings from three high-quality studies
Moderate	Consistent findings from at least one high-quality and one or more low-quality studies
Limited	Consistent findings in one low-quality study or only one study available
Conflicting	Inconsistent evidence in multiple studies, irrespective of study quality
No evidence	No studies found

The ICC and Pearson’s correlation coefficient were interpreted according to Munro and Visintainer [36] as follows: (i) Very low correlation: .00-.29; (ii) Low correlation: .30-.49; (iii) Moderate correlation: .50-.69; (iv) High correlation: .70-.89; (v) Very high correlation: .90-1.00.

Results

Selection of Studies

**Figure 1:**
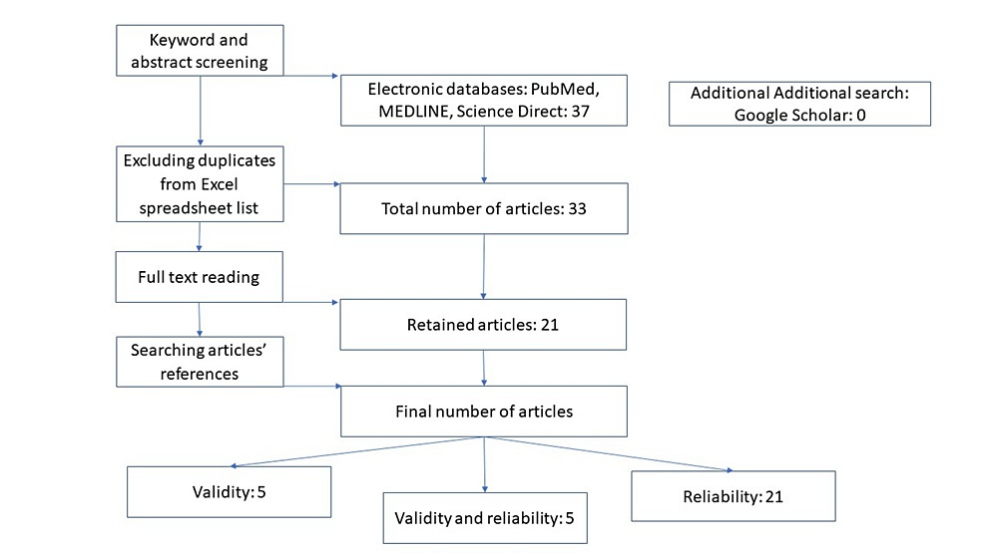
PRISMA flow diagram PRISMA: Preferred Reporting Items for Systematic Reviews and Meta-Analyses

In total, 21 articles were reviewed based on the selection criteria mentioned above. Of these, five studies examined validity and reliability, and 17 evaluated only reliability. Of the 21 reliability studies, 15 investigated both intra- and inter-rater reliability, five investigated only intra-rater reliability, and one investigated only inter-rater reliability. Figure 1 presents the Preferred Reporting Items for Systematic Reviews and Meta-Analyses (PRISMA) diagram describing the selection process of articles [37].

Methodological Quality

Fourteen of the 21 studies were deemed of high quality. The full scoring is given in Table 2. After discussion and deliberation, the two reviewers agreed on the scores to be attributed. All five studies that examined validity were of high quality [38-42].

**Table 2:** Methodological quality of studies 1: Adequate description of the study population; 2: Adequate description of raters; 3: Adequate explanation of reference standard; 4: Between-rater blinding; 5: Within-rater blinding; 6: Variation of testing order; 7: Time between index test and reference standard; 8: Time between repeated measures; 9: Independency of reference standard from index test; 10: Adequate description of index test procedure; 11: Adequate description of reference standard procedure; 12: Explanation of any withdrawals; 13: Appropriate statistical methods. N/A: not applicable

Study	1	2	3	4	5	6	7	8	9	10	11	12	13	High quality?
Gadotti et al., 2013 [17]	Yes	Yes	Yes	Yes	Yes	Yes	Yes	Yes	No	Yes	Yes	N/A	Yes	Yes
Garrett et al., 1993 [18]	Yes	Yes	No	Yes	Yes	Yes	N/A	Yes	N/A	Yes	N/A	No	Yes	Yes
Hickey et al., 2000 [20]	Yes	Yes	Yes	Yes	Yes	Yes	Yes	Yes	Yes	Yes	Yes	No	Yes	Yes
Gallego-Izquierdo et al., 2020 [38]	Yes	Yes	N/A	Yes	Yes	Yes	N/A	Yes	N/A	Yes	N/A	No	Yes	Yes
Hopkins et al., 2019 [39]	Yes	Yes	Yes	N/A	N/A	Yes	Yes	Yes	Yes	Yes	Yes	No	Yes	Yes
Lau et al., 2010 [40]	Yes	Yes	Yes	Yes	Yes	Yes	Yes	Yes	Yes	Yes	Yes	No	Yes	Yes
Ruivo et al., 2013 [41]	N/A	Yes	Yes	Yes	Yes	Yes	N/A	Yes	Yes	Yes	Yes	No	Yes	Yes
van Niekerk et al., 2008 [42]	Yes	Yes	Yes	N/A	N/A	No	Yes	Yes	No	Yes	Yes	N/A	Yes	Yes
Dimitriadis et al., 2015 [43]	Yes	Yes	N/A	Yes	No	Yes	N/A	Yes	N/A	Yes	N/A	N/A	Yes	No
Dunk et al., 2005 [44]	Yes	No	N/A	N/A	Yes	N/A	Yes	N/A	N/A	Yes	N/A	N/A	Yes	No
Gadotti et al., 2010 [45]	Yes	Yes	Yes	N/A	No	No	No	No	Yes	Yes	Yes	N/A	Yes	No
Moradi et al., 2014 [46]	Yes	Yes	N/A	Yes	Yes	Yes	No	Yes	No	Yes	No	No	Yes	Yes
Nam et al., 2013 [47]	Yes	Yes	N/A	Yes	Yes	Yes	N/A	Yes	N/A	Yes	No	No	Yes	Yes
Salahzadeh et al., 2014 [48]	Yes	Yes	N/A	N/A	N/A	Yes	Yes	Yes	N/A	Yes	N/A	No	Yes	No
Ferreira et al., 2010 [49]	No	Yes	Yes	Yes	Yes	Yes	Yes	Yes	Yes	Yes	Yes	N/A	Yes	Yes
Ruivo et al., 2015 [50]	Yes	Yes	Yes	Yes	Yes	Yes	Yes	Yes	N/A	Yes	No	Yes	Yes	Yes
Souza et al., 2011 [51]	Yes	No	N/A	N/A	Yes	Yes	N/A	Yes	N/A	Yes	N/A	No	Yes	No
Weber et al., 2012 [52]	Yes	Yes	Yes	N/A	N/A	Yes	Yes	Yes	Yes	Yes	Yes	N/A	Yes	Yes
Cote et al., 2021 [53]	Yes	Yes	N/A	Yes	Yes	No	N/A	Yes	N/A	Yes	N/A	N/A	Yes	No
Gray et al., 2020 [54]	Yes	Yes	No	Yes	Yes	Yes	No	Yes	N/A	Yes	No	No	Yes	Yes
Lee et al., 2017 [55]	Yes	No	N/A	No	No	Yes	N/A	Yes	N/A	Yes	N/A	N/A	Yes	No

Study Characteristics

Eleven different methods of measuring FHP were identified within the reviewed articles. The most studied were photogrammetry and Postural Assessment Software (SAPO version 0.69). Classic photogrammetry was not tested for validity. SAPO’s validity was examined in only one study [41]. The full list of methods is given in Table 3.

**Table 3:** List of methods FHP: forward head posture; CROM: cervical range of motion

1. Photogrammetry: Seven articles [17, 43-48]
2. Postural Assessment Software (SAPO): Five articles [41,49-52]
3. Photogrammetry through Video Conferencing Platform: One article [53]
4. Digital Photogrammetry with PostureScreen Mobile App (PostureCo, Inc., Florida, United States): One article [39]
5. FHP App (Pyeongtaek, South Korea) Mobile Phone Application: One article [38]
6. Goniometer, CROM Instrument: One article [18]
7. Goniometer, Posture Measuring Device (PMD): One article [54]
8. Goniometer, CROM Device and Plumb-Line Techniques: One article [20]
9. Goniometer, SmartTool Angle Finder (M-D Building Products, Inc., Oklahoma City, United States): One article [40]
10. 3D Motion Capture System: Ibe article [55]
11. Photographic Posture Analysis Method (PPAM): One article [42]

Types of Participants

A healthy population was included in 12 of 21 studies [20,38,39,42,43-48,50,54]. A mixed population of healthy and neck-pain participants was included in four of 21 studies [18,40,51,53]. Five of 21 studies did not report on the health condition of participants [41,45,49,52,55]. The participants’ BMI was reported in 10 out of 21 studies [17,18,38,39,43,44-46,53,55].

The population age varied between studies. Underage populations aged 15-16 and 16-17 years took part in two studies [42,50]. Two studies’ populations were young adults aged 18-28 and 17-27 years [17,45]. The population of one study was between the ages of 19 and 35 years [52]. Seven studies included participants who were predominantly in their early twenties, within the age group of 18-27 years [38,39,43,44,47,48,55]. Three studies included a population aged 25-26 years [20,51,53]. Three studies included older populations of 33 ± 8.03 years [45], 46.7 ± 9.5 years (40) and 50 ± 15.7 years [18]. Finally, three studies did not report the population age [41,49,54].

Reliability and Validity

The validity of the methods ranged from low to very high. However, only five of 21 studies assessed validity. Reliability showed varying results, given that not all studies investigated both inter- and intra-rater reliability. More detailed results are shown in Table 4.

**Table 4:** Reliability and validity data for all methods ICC: intraclass correlation coefficient; CROM: cervical range of motion; SEM: standard error of measurement; N/A: not applicable

Reference	High quality?	Reliability (ICC/Cronbach’s alpha)	SEM	Validity (correlation coefficient)
Gadotti and Magee, 2013 [17]	Yes	.99 (inter), .99 (intra)	0.04 (inter), 0.01 (intra)	N/A
Garrett et al., 1993 [18]	Yes	.93 (intra), .83 (inter)	N/A	N/A
Hickey et al., 2000 [20]	Yes	CROM 0.77 (intra) 0.69 (inter), plumb line 0.83 (intra), 0.75 (inter)	N/A	N/A
Gallego-Izquierdo et al., 2020 [38]	Yes	.86 (intra), 0.88 (inter)	1.96 (intra), 1.795 (inter)	0.86 (correlation coefficient)
Hopkins et al., 2019 [39]	Yes	1.00 ± 0.09 (intra)	N/A	-0.14 ± 0.06 (-0.32; 0.04) bias (99.75% credible Interval)
Lau et al., 2010 [40]	Yes	.99 (inter), 0.99 (intra)	N/A	0.72 (correlation coefficient)
Ruivo et al., 2013 [41]	Yes	.99 (inter), 0.99 (intra)	N/A	0.94 (correlation coefficient)
van Niekerk et al., 2008 [42]	Yes	.98 (inter)	8.06	0.89 (correlation coefficient)
Dimitriadis et al., 2015 [43]	No	Sitting .86, standing .82 (inter), sitting .86, standing .88 (intra)	Sitting 1.7, standing 1.94 (inter), sitting 2.08, standing 1.75 (intra)	N/A
Dunk et al., 2005 [44]	No	.83–.74 (intra)		N/A
Gadotti et al., 2010 [45]	No	.85 (intra)		N/A
Moradi et al., 2014 [46]	Yes	.95 (intra), .89 (inter)	0.74 (intra), 1.5 (inter)	N/A
Nam et al., 2013 [47]	Yes	.75 (inter), 0.91 (intra)	0.13 (inter), 0.16 (intra)	N/A
Salahzadeh et al., 2014 [48]	No	.90 (inter), 0.92 (intra)	1.94 (inter), 1.74 (intra)	N/A
Ferreira et al., 2010 [49]	Yes	.69 (inter), .85 (intra)	1.77 (inter), 1.33 (intra)	N/A
Ruivo et al., 2015 [50]	Yes	.88 (inter), 0.83 (intra)	2.35 (inter), 2.72 (intra)	N/A
Souza et al., 2011 [51]	No	.99 (ANOVA-p-intra), .98 (inter)	N/A	N/A
Weber et al., 2012 [52]	Yes	.99 (intra)		N/A
Cote et al., 2021 [53]	No	.88 (inter), 0.91 (intra)	1.87 (inter), 1.89 (intra)	N/A
Gray et al., 2020 [54]	Yes	.82 (intra) 0.87 (inter)	N/A	N/A
Lee et al., 2017 [55]	No	Sitting .92, standing .96 (intra)		N/A

Level of Evidence

Table 5 details the accumulated levels of evidence found for all examined methods. For most studies, there was a limited or inconsistent level of evidence for the reliability and validity of the methods. Strong and moderate levels of evidence were found for a small selection of methods.

**Table 5:** Level of evidence FHP: forward head posture; CROM: cervical range of motion; PPAM: photographic posture analysis method; PMD: posture measuring device

Level of evidence	Method	Reliability	Validity
Strong	Photogrammetry	Good to excellent intra-rater and inter-rater reliability	
	SAPO software	Good to excellent intra-rater and inter-rater reliability	
	FHP app (Pyeongtaek, South Korea) mobile phone application	Very good intra- and inter-rater reliability	Very high validity
	Goniometer, CROM instrument	Very good intra- and inter-rater reliability	
	Goniometer, CROM device and plumb-line techniques	Moderate intra- and inter-rater reliability	
	PPAM	Excellent intra-rater reliability	Very high validity
	Goniometer, SmartTool Angle Finder (M-D Building Products, Inc., Oklahoma City, United States)	Excellent intra- and inter-rater reliability	Moderate validity
Moderate	Photogrammetry	Good to excellent intra-rater and inter-rater reliability	
	Goniometer, PMD	Very good intra- and inter-rater reliability	
	Digital photogrammetry with PostureScreen Mobile App (PostureCo, Inc., Trinity, FL, United States)	Excellent intra-rater reliability	High validity
	3D Motion Capture System	Excellent intra-rater reliability	
	SAPO software	Excellent intra-rater and inter-rater reliability	
Limited	SAPO software	Excellent intra-rater and inter-rater reliability	Very high validity

Discussion

Main Findings

This systematic review examined 11 methods for the non-invasive measurement of FHP, excluding radiography. These included a variety of approaches, from classic photogrammetric methods measuring the CVA to digital postural assessment tools and mobile applications. Levels of reliability varied significantly between methods. However, it is not feasible to draw safe conclusions because of the limited number of references per method. An adequate number of references examined were present for classic photogrammetry and postural assessment software (seven and five, respectively). Both methods ranked a score of good to excellent intra-rater and inter-rater reliability. Digital photogrammetry’s reliability was studied in only two articles; this was done using two different tools, a video conferencing article [53] and a mobile application [39]. Goniometry as a method was studied in five articles using five different instruments. Upon reviewing the data, it was found that classic photogrammetry and postural assessment software seem to be equally reliable.

The validity of measurement methods has been less commonly studied. However, photogrammetry as a method is generally accepted as valid.

Validity

Spinal X-rays are considered the gold standard method for assessing spinal deformities, including such postural alterations as FHP. However, radiographs impose accessibility and ethical obstacles on different populations [56]. This is why only one study examined photogrammetry versus radiography [42], and another one study examined goniometry versus radiography [39]. In addition, the placement of surface landmarks used to locate the tragus, and C7 cannot be considered to be as accurate as locating those spots on radiographic images.

Gallego-Izquierdo et al. [38] found high criterion validity of the FHP app using photogrammetry via a software program (Kinovea) that automatically calculates CVA [39]. The study's very good intra- and inter-rater reliability also supported its internal validity. Lau et al. [40] showed good criterion validity of the SmartTool Angle Finder (M-D Building Products, Inc., Oklahoma City, United States) goniometer against X-rays. The study's excellent intra- and inter-rater reliability also supported its internal validity.

Van Niekerk et al. showed good criterion validity of computerised photogrammetry of the PPAM method against radiography using the LODOX system (Lodox Systems (Pty) Ltd, Sandton, South Africa) [42]. Ruivo et al. showed high validity of the SAPO method examining classic goniometry [41]. However, the level of evidence was limited; therefore, goniometry is not considered a solidly valid method for FHP measurement. The excellent intra- and inter-rater reliability of the study supported its internal validity.

Hopkins et al. [39] performed the first study to evaluate the validity of digital photogrammetry with the PostureScreen Mobile App (PostureCo, Inc., Trinity, FL, United States), but the results were uncertain. In addition, the level of evidence of the study was moderate. 

Based on the above, no definitive conclusions can be drawn regarding the validity of the assessment of FHP by non-invasive techniques other than radiographic assessment. Although there are indications that photogrammetry can produce valid results in assessing FHP, this should be confirmed by future validity studies. Until then, the radiographic evaluation will remain essential in the clinical evaluation of FHP.

 Reliability

The reliability of measurement methods depends heavily on eliminating the uncertainty caused by postural discrepancies. The vast majority of researchers attempted to address this point via variation of the testing order (except for four studies) [42,44,45,53] and by taking measurements in repeated periods (except for two studies) [44,45].

Measurement of CVA required accurately locating the relevant spots on the anatomy-for example, the C7 and tragus for photogrammetry-and in some cases, the placing of surface landmarks. Therefore, reliability depends highly on accuracy. Twenty studies described the procedure followed to improve accuracy in detail; the one study that did not do so was that of Gadotti et al. [45]. In addition, 10 studies included experienced testers [18,20,38,40,45,47,48,50,53,54]. Moreover, only two studies took measurements in both sitting and standing positions [43,55].

Based on the above, it can be concluded that non-invasive evaluation techniques of the FHP can produce reliable results if the measurement process is standardized. However, the articles' significant methodological differences and characteristics (high heterogeneity of study populations, measurement methods, and raters) make conclusions generalization difficult. Despite this, there were a sufficient number of reports documenting the reliability of these techniques for assessing FHP, particularly using classical photogrammetry and postural assessment software (PAS/SAPO).

Methodological Considerations

The methodological limitations of the reviewed studies involved the general health and condition of the studied populations. Most included a healthy population sample with a mean age of between 20 and 65 years. The BMI was unreported in 11 of the 21 studies. These characteristics do not accurately and inclusively reflect the patients who will receive FHP measurements in clinical practice [29]. Therefore, the results for diagnostic accuracy may have limited clinical applicability (generalisability).

Raters were described in only three out of the five SAPO-related studies, calling into question the possibility of generalising results for this method. Moreover, some studies did not perform inter-rater blinding [39,42,44,45,48,51,52,55] or intra-rater blinding [39,43,42,45,48,52,55].

Limitations of the Review

The present review was conducted in a systematic manner, incorporating the PRISMA guidelines for the search of studies and QUADAS and QAREL for qualitatively assessing their methodology. In addition, two reviewers were engaged, as well as all available populations. However, the review had limitations in that it included only articles from English and Greek language. Moreover, two reviewers had knowledge of the results of the studies before assessing their methodological quality. The critical appraisal tool was applied with the strictest criteria to limit the possibility of reviewer bias [57]. Finally, the high heterogeneity of study populations, measurement methods, and raters suggests that the external validity of this scoping review is low.

Clinical and Research Implications

The examined methods showed that therapists could choose a method to assess FHP from a limited number of approaches. The most widespread methods are radiography, classic photogrammetry and goniometry.

Photogrammetry can be recommended as a reliable and valid method to use without the disadvantages of radiography. Digital photogrammetry is trending, and different software and mobile applications have been tested with limited data so far. It may be useful for specific populations to make use of video-based telehealth platforms, as examined in Cote et al.’s study [53]. Goniometry is a widespread approach but it is performed with different instruments, leaving the therapist to decide which is most suitable for them; thus, there are no conclusive outcomes as to the reliability and validity of each instrument. Experience in the use of each goniometer is also a determining factor for measurement accuracy. Further research could inform the use of the appropriate goniometer.

It can be stated from this review that therapists need to consider population characteristics when deciding on the appropriate FHP assessment method. In addition, future research should include more representative samples of populations, ensure rater blinding and focus on appropriate statistical analyses.

## Conclusions

This systematic review examined various FHP measurement methods, including photogrammetry, postural assessment software, mobile applications, goniometer measurements, and 3D motion capture systems. However, the number of studies examining each method was limited except for photogrammetry. Overall, the reliability data were positive, but such data remain limited; in some cases, the data presented significant limitations. Photogrammetry consistently delivers reliable results. In contrast, the different goniometers used in goniometry methods do not allow a definite conclusion regarding the method’s overall reliability. Validity data are very limited throughout the methods, although photogrammetry appears to be considered valid. Ultimately, further research is needed to evaluate the reliability and validity of goniometry, solidify the validity of photogrammetry, and provide data on other reviewed methods.
